# Education, MoCA‐Defined Baseline Cognitive Status, and Screening‐Level Cognitive Change After Bilateral Subthalamic Nucleus Deep Brain Stimulation in Parkinson’s Disease: A Retrospective Cohort Study

**DOI:** 10.1155/padi/1801194

**Published:** 2026-11-14

**Authors:** Siyan Chen, Yuting Zhang, Chenchen Cui, Min Xu, Wei Zhang, Dayong Shen, Jie Zu

**Affiliations:** ^1^ Department of Neurology The Affiliated Hospital of Xuzhou Medical University Xuzhou Jiangsu China xzmc.edu.cn

**Keywords:** cognition, deep brain stimulation, education, MoCA, Parkinson’s disease, subthalamic nucleus

## Abstract

**Background:**

Cognitive outcomes after subthalamic nucleus deep brain stimulation (STN‐DBS) in Parkinson’s disease (PD) remain variable. Whether education, a proxy for cognitive reserve, is associated with postoperative MoCA‐based cognitive performance is uncertain.

**Methods:**

This retrospective cohort included 111 patients with PD who underwent bilateral STN‐DBS from 2015 to 2024 and had paired Montreal Cognitive Assessment (MoCA) data. Patients were classified as MoCA‐defined mild cognitive impairment (MCI; baseline MoCA ≤ 21; *n* = 73) or normal baseline cognition (MoCA > 21; *n* = 38). Unadjusted MoCA change was described, and postoperative MoCA performance was evaluated using baseline‐adjusted multiple linear regression. Clinically meaningful decline was defined as a MoCA decrease of at least 2 points, and sensitivity analyses additionally assessed the influence of postoperative follow‐up duration.

**Results:**

In unadjusted analyses, mean MoCA increased modestly overall (+0.9 ± 4.5; *p* = 0.012). The unadjusted increase was greater in the MoCA‐defined MCI group than in the normal‐cognition group (+1.7 ± 4.6 vs. −0.5 ± 3.8; *p* = 0.020), but the cognitive‐status group was not associated with postoperative MoCA after adjustment for baseline MoCA (*B* = 0.683, *p* = 0.627). Clinically meaningful cognitive decline occurred in 23.4%. Higher education was associated with higher postoperative MoCA performance (*B* = 0.514, *p* < 0.001) and lower odds of decline in the full (odds ratio [OR] = 0.751, *p* = 0.005) and parsimonious (OR = 0.776, *p* = 0.003) models. The education‐related associations remained materially unchanged in sensitivity analyses, additionally adjusting for follow‐up duration.

**Conclusions:**

Group‐level MoCA‐based cognitive screening performance showed no overall deterioration after bilateral STN‐DBS. The greater unadjusted increase among patients with lower baseline MoCA scores should be interpreted cautiously because baseline‐score effects may contribute. Education may provide useful adjunctive information for cognitive risk assessment but cannot replace comprehensive neuropsychological evaluation.

## 1. Introduction

Cognitive impairment is a common and clinically important nonmotor manifestation of Parkinson’s disease (PD) [1]. Mild cognitive impairment (MCI) affects approximately 40% of nondemented patients with PD, and its prevalence increases with age and disease duration [2]. Cognitive decline reduces independence, worsens quality of life, and complicates long‐term treatment planning. Subthalamic nucleus deep brain stimulation (STN‐DBS) is an established surgical treatment for advanced PD, providing sustained motor benefit and reducing dopaminergic medication burden [3]. Its cognitive consequences, however, are less predictable.

Long‐term neuropsychological studies suggest that the cognitive effects of DBS are usually domain‐specific rather than global [4, 5]. In a meta‐analysis of 48 DBS studies, the most consistent postoperative declines involved verbal fluency, delayed recall, and selected executive measures, whereas global cognition was generally less affected [6]. At the individual‐patient level, clinically meaningful cognitive decline after STN‐DBS has still been reported in 10%–40% of cases, depending on the assessment instrument, follow‐up interval, and decline threshold [7, 8]. This uncertainty is clinically relevant for surgical selection, as patients with low preoperative screening scores are often considered cognitively vulnerable. However, cognitive impairment before DBS is not necessarily synonymous with postoperative decline; in a recent cohort study, patients with PD‐MCI had a lower risk of Montreal Cognitive Assessment (MoCA)‐defined decline than cognitively normal patients [9].

The predictors of post‐DBS cognitive change remain difficult to translate into individual risk estimates. Recent reviews have identified older age and poorer preoperative neuropsychological status as the most reproducible risk markers, whereas reserve‐related variables have been studied less consistently. In this context, education is a clinically practical candidate marker. It is routinely available, imposes no additional testing burden, and is commonly used as a proxy for cognitive reserve, a construct that describes the capacity to maintain cognitive performance despite neuropathological burden [10, 11]. Longitudinal PD cohorts have linked higher educational attainment and broader cognitive‐reserve measures to slower cognitive decline or better cognitive performance [12–14], although whether this reserve‐related association is also detectable after DBS remains uncertain [15, 16].

Against this background, we examined MoCA‐based cognitive screening outcomes after bilateral STN‐DBS, with prespecified stratification by baseline MoCA‐defined cognitive status. We also evaluated whether years of education were independently associated with postoperative MoCA performance and clinically meaningful MoCA decline after accounting for baseline cognitive performance and other clinical variables. We hypothesized that higher educational attainment would be associated with more favorable postoperative MoCA‐based cognitive outcomes.

## 2. Methods

### 2.1. Study Design and Ethics

We conducted a single‐center retrospective cohort study at the Department of Neurology, Parkinson’s Disease Center, The Affiliated Hospital of Xuzhou Medical University, Xuzhou, China. Medical records were reviewed for all patients with idiopathic PD who underwent bilateral STN‐DBS between January 2015 and December 2024. The study was approved by the Ethics Committee of the Affiliated Hospital of Xuzhou Medical University (approval no. XYFY2024‐KL501‐01). The study was conducted in accordance with the Declaration of Helsinki and reported according to the Strengthening the Reporting of Observational Studies in Epidemiology (STROBE) guidelines. Patient privacy and confidentiality were protected throughout the study.

### 2.2. Patient Selection

Patients were eligible if they had idiopathic PD diagnosed according to the United Kingdom Parkinson’s Disease Society Brain Bank criteria, underwent bilateral STN‐DBS during the study period, had a preoperative MoCA score within 1 month before surgery, had at least one routine postoperative MoCA assessment at least 3 months after surgery, and were aged ≥ 18 years at surgery.

Patients were excluded if they had atypical or secondary Parkinsonism, previous neurosurgical treatment for PD, severe psychiatric comorbidity precluding reliable cognitive testing, incomplete or unreliable cognitive data, or DBS lead revision or explantation before postoperative cognitive assessment.

### 2.3. Neurosurgical Procedures

All patients underwent bilateral STN‐DBS performed by the same multidisciplinary DBS team using a standardized frame‐based stereotactic protocol. Preoperative MRI and CT images were fused in the surgical planning system to localize the bilateral STN and plan electrode trajectories. After application of a Leksell stereotactic frame (Elekta Instrument AB, Stockholm, Sweden) under local anesthesia, microelectrode recording and, when clinically indicated, macrostimulation were used to verify the target and select the final trajectory. Quadripolar DBS electrodes (model 3389; Medtronic, Dublin, Ireland) were implanted bilaterally. Extension leads and the implantable pulse generator were implanted in a second stage procedure under general anesthesia. Postoperative CT was obtained within 1–3 days to confirm lead location and exclude intracranial hemorrhage. The stimulator was usually activated approximately 1 month after surgery, followed by routine programming and medication optimization during follow‐up.

### 2.4. Clinical Assessments

Cognitive assessment. The cognitive outcome measure was the Beijing version of the MoCA, a 0–30‐point global cognitive screening instrument. The MoCA was administered within 1 month before surgery and at the latest available routine postoperative follow‐up at least 3 months after surgery, after DBS activation and clinical stabilization whenever feasible, by the same movement disorders team. The postoperative cognitive follow‐up interval was calculated as the time from DBS surgery to the postoperative MoCA assessment used in the analysis. In the analytic cohort, the median interval from DBS surgery to postoperative MoCA assessment was 11.4 months (interquartile range [IQR], 10.4–13.3; range, 5.2–40.8 months). Domain‐level MoCA subscores were not systematically available in the retrospective records. Complete paired pre‐ and postoperative domain‐level data were available for only three patients in the final cohort, precluding reliable domain‐specific analyses.

Motor assessment. Motor function was assessed using Part III of the Unified Parkinson’s Disease Rating Scale (UPDRS‐III). Preoperative assessments were obtained in medication‐off and medication‐on states; the medication‐off state was defined as withdrawal of dopaminergic medication for at least 12 h. Postoperative UPDRS‐III scores were recorded in four conditions when available: DBS‐off/medication‐off, DBS‐on/medication‐off, DBS‐off/medication‐on, and DBS‐on/medication‐on. For postoperative DBS‐off assessments, stimulation was switched off before testing according to the center’s routine clinical protocol, which included a predefined waiting period. However, the exact switch‐off time and achieved washout duration were not consistently documented in the retrospective records and therefore could not be verified for every assessment. The levodopa equivalent daily dose (LEDD) was calculated using established conversion factors. Motor outcomes were analyzed as descriptive background measures rather than primary efficacy outcomes and are reported in the supporting information (available here).

Other assessments. Additional preoperative variables included the Beck Depression Inventory score, the Hoehn–Yahr stage in the medication‐off state, the Parkinson’s Disease Questionnaire‐39 score, and years of formal education. Education level was categorized as primary school or below (≤ 6 years), middle school (7–9 years), or high school and above (≥ 10 years).

### 2.5. Outcome Definitions and Group Classification

The primary outcome was the change in MoCA total score from preoperative baseline to postoperative follow‐up, calculated as postoperative MoCA minus preoperative MoCA. Secondary outcomes included clinically meaningful MoCA decline and MoCA change stratified by baseline cognitive status. Clinically meaningful cognitive decline was defined as a MoCA decrease of at least 2 points.

Baseline cognitive status was defined according to the preoperative MoCA assessment. Patients with MoCA scores ≤ 21 were classified as having MoCA‐defined MCI, whereas those with MoCA > 21 were classified as having normal baseline cognition. This definition was an operational screening‐based classification, not a formal diagnosis of PD‐MCI based on comprehensive neuropsychological criteria [9, 15]. Sensitivity analyses used alternative baseline MoCA cutoffs of ≤ 20 and ≤ 22. An additional education‐adjusted sensitivity analysis added 1 point to the baseline MoCA for patients with ≤ 12 years of education, capped the adjusted score at 30, and then applied the ≤ 21 cutoff.

### 2.6. Statistical Analysis

Continuous variables were summarized as mean ± standard deviation (SD) or median with IQR, as appropriate; categorical variables were summarized as counts and percentages. Normality was assessed using the Shapiro–Wilk test. Baseline characteristics were compared with the Mann–Whitney *U* test for continuous variables and the chi‐square or Fisher’s exact test for categorical variables. To evaluate potential selection bias related to postoperative cognitive follow‐up, baseline characteristics were also compared between included and excluded patients. The interval from DBS surgery to the postoperative MoCA assessment used for analysis was calculated in months and summarized using the median, IQR, and range. Follow‐up duration was compared between the MoCA‐defined MCI and normal‐cognition groups using the Mann–Whitney *U* test.

Pre‐ and postoperative cognitive scores were compared using the two‐sided Wilcoxon signed‐rank test. Effect sizes were calculated as $r=\left|Z\right|/\sqrt{\left(N\right)}$. Exploratory subgroup analyses of MoCA change were performed by baseline cognitive status, age group, education level, sex, and disease duration. Between‐group differences in MoCA change were tested with the Mann–Whitney *U* test.

Adjusted associations with postoperative cognitive performance were evaluated using baseline‐adjusted multiple linear regression, with postoperative MoCA score as the dependent variable and baseline MoCA score included as a covariate. Additional covariates were age at surgery, sex, disease duration, UPDRS‐III medication‐off score, LEDD, Beck Depression Inventory score, and years of education. To address potential baseline‐score dependence and regression to the mean in the unadjusted comparison of MoCA change between cognitive‐status groups, an additional baseline‐adjusted sensitivity analysis modeled postoperative MoCA as a function of baseline MoCA score and MoCA‐defined cognitive‐status group. Binary logistic regression was used to identify predictors of clinically meaningful cognitive decline. The full model included the same covariates as the multivariable linear model. Because the number of decline events was limited, we also fitted a parsimonious model prespecified to include baseline MoCA, education, and age to reduce overfitting risk. Regression analyses were performed using complete cases for the variables included in each model. Results are reported as unstandardized regression coefficients (B) or odds ratios (ORs) with 95% confidence intervals (CIs).

Because the timing of postoperative cognitive assessment varied in routine clinical practice, sensitivity analyses additionally included follow‐up duration as a covariate to assess whether assessment timing materially influenced the main findings. Follow‐up duration was added to the baseline‐adjusted linear model, the baseline cognitive‐status sensitivity model, and the parsimonious logistic model for clinically meaningful MoCA decline. The parsimonious logistic model was used for this sensitivity analysis to limit overfitting given the number of decline events.

All tests were two‐sided, with statistical significance set at *α* = 0.05. Subgroup analyses were exploratory; no formal adjustment for multiple comparisons was applied, and exact *p* values are reported to support cautious interpretation. Analyses were primarily performed using R software, Version 4.4.2, with assistance from JD_DCPM software, Version 6.11 (Jingding Medical Technology Co., Ltd., China), for data processing, statistical implementation, and visualization.

## 3. Results

### 3.1. Patient Characteristics

During the study period, 280 patients underwent bilateral STN‐DBS. After exclusion of 169 patients, including 157 without postoperative MoCA assessment and 12 with incomplete cognitive data, 111 patients with paired pre‐ and postoperative MoCA scores were included in the final analysis. The patient selection process is shown in Figure 1. Sex data were available for 110 patients, of whom 62 (56.4%) were men. Mean age at surgery was 62.9 ± 8.1 years, mean disease duration was 9.0 ± 4.6 years, and mean formal education was 8.4 ± 4.0 years. Baseline MoCA, education, depression score, and Parkinson’s Disease Questionnaire‐39 score were similar between included and excluded patients. Compared with excluded patients, included patients underwent surgery earlier and had slightly better medication‐on‐motor scores and levodopa responsiveness (Supporting Table S2).

**FIGURE 1:**
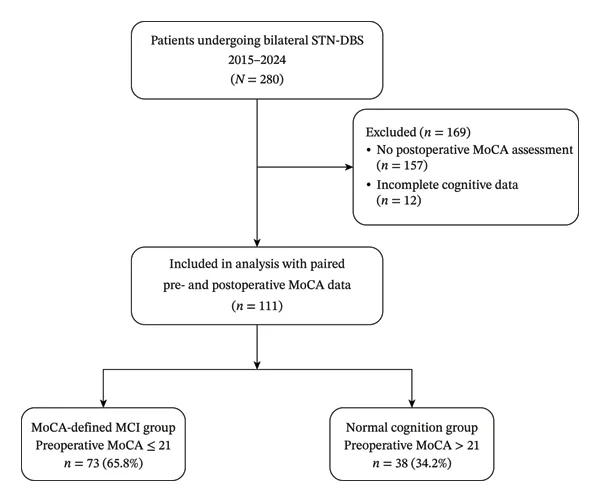
Patient selection flowchart showing patient identification and selection for the final cognitive analysis. Of 280 patients who underwent bilateral STN‐DBS between 2015 and 2024, 169 were excluded, including 157 without postoperative MoCA assessment and 12 with incomplete cognitive data. The final cohort included 111 patients with paired pre‐ and postoperative MoCA data. Abbreviations: DBS, deep brain stimulation; MCI, mild cognitive impairment; MoCA, Montreal Cognitive Assessment; STN, subthalamic nucleus.

Using the preoperative MoCA cutoff, 73 patients (65.8%) were classified as having MoCA‐defined MCI and 38 (34.2%) as having normal baseline cognition. Compared with the normal cognition group, the MoCA‐defined MCI group was older (64.2 ± 6.5 vs. 60.4 ± 10.1 years; *p* = 0.047), had a longer disease duration (9.7 ± 5.0 vs. 7.6 ± 3.1 years; *p* = 0.018), higher UPDRS‐III medication‐off scores (62.1 ± 18.4 vs. 50.2 ± 17.0; *p* = 0.004), higher Beck Depression Inventory scores (21.6 ± 11.3 vs. 14.7 ± 8.2; *p* = 0.003), and fewer years of education (7.4 ± 4.0 vs. 10.4 ± 3.4; *p* < 0.001). Sex distribution and LEDD did not differ significantly between groups (Table 1).

**TABLE 1:** Baseline demographic and clinical characteristics stratified by baseline cognitive status.

Characteristic	Overall (*n* = 111)	MoCA‐defined MCI (*n* = 73)	Normal cognition (*n* = 38)	*p*
*Demographic characteristics*				
Male sex, *n* (%)	62 (56.4%)	38 (52.8%)	24 (63.2%)	0.400
Age at surgery, years	62.9 ± 8.1	64.2 ± 6.5	60.4 ± 10.1	0.047
Disease duration, years	9.0 ± 4.6	9.7 ± 5.0	7.6 ± 3.1	0.018

*Clinical characteristics*				
UPDRS‐III medication‐off	58.0 ± 18.7	62.1 ± 18.4	50.2 ± 17.0	0.004
UPDRS‐III medication‐on	28.5 ± 13.1	31.2 ± 12.7	23.3 ± 12.4	0.003
Levodopa response, %	51.3 ± 14.1	49.3 ± 13.3	55.0 ± 15.1	0.060
LEDD, mg/day	772.8 ± 342.0	789.6 ± 353.3	738.2 ± 320.0	0.625
Hoehn–Yahr stage, medication‐off	2.9 ± 0.7	3.0 ± 0.7	2.8 ± 0.7	0.356

*Cognitive, mood, and patient-reported measures*				
Baseline MoCA	19.2 ± 4.8	16.4 ± 3.3	24.4 ± 2.4	< 0.001
BECK depression score	19.3 ± 10.8	21.6 ± 11.3	14.7 ± 8.2	0.003
PDQ‐39 score	61.8 ± 27.1	66.5 ± 27.8	52.2 ± 23.0	0.003

*Education*				
Education, years	8.4 ± 4.0	7.4 ± 4.0	10.4 ± 3.4	< 0.001
Education level, *n* (%)				0.004
Primary (≤ 6 years)	33 (31.1%)	28 (40.6%)	5 (13.5%)	
Middle school (7–9 years)	44 (41.5%)	28 (40.6%)	16 (43.2%)	
High school or above (≥ 10 years)	29 (27.4%)	13 (18.8%)	16 (43.2%)	

*Note:* Values are mean ± SD or *n* (%), unless otherwise indicated. MoCA‐defined MCI was operationally defined as baseline MoCA ≤ 21; normal cognition was defined as baseline MoCA > 21. *p* values compare the MoCA‐defined MCI and normal cognition groups and were calculated using the Mann–Whitney *U* test or chi‐square test, as appropriate. Denominators varied because of missing data. Education‐level percentages were calculated among patients with available education data. BECK, Beck Depression Inventory; MoCA, Montreal Cognitive Assessment; UPDRS‐III, Unified Parkinson’s Disease Rating Scale Part III.

Abbreviations: LEDD, levodopa equivalent daily dose; MCI, mild cognitive impairment; PDQ‐39, Parkinson’s Disease Questionnaire‐39.

The median interval from DBS surgery to postoperative MoCA assessment was 11.4 months (IQR, 10.4–13.3; range, 5.2–40.8 months). Follow‐up duration was similar between patients with MoCA‐defined MCI and those with normal baseline cognition [median 11.3 months (IQR, 10.3–13.2) vs. 11.5 months (IQR, 10.6–13.5), respectively; *p* = 0.666].

### 3.2. Motor Outcomes

Motor outcomes were analyzed as descriptive background measures in the broader surgical cohort with paired UPDRS‐III data. The UPDRS‐III medication‐off score was 58.4 ± 16.9 preoperatively, compared with 25.9 ± 13.1 postoperatively in the DBS‐on/medication‐on condition, corresponding to a 54.8% reduction in motor score (*p* < 0.001). In the DBS‐off/medication‐off comparison, the postoperative score was 48.8 ± 19.6 compared with 57.9 ± 16.9 preoperatively, corresponding to a 14.0% reduction (*p* < 0.001). This finding should be interpreted descriptively because the exact stimulation washout duration could not be verified for all retrospective assessments. Additional motor‐state comparisons are shown in Supporting Table S1.

### 3.3. Overall Cognitive Outcomes

In the overall cohort, MoCA scores increased from 19.2 ± 4.8 before surgery to 20.1 ± 5.7 after surgery (Δ = +0.9 ± 4.5; *p* = 0.012; *r* = 0.238) (Table 2). Overall, 59 patients (53.2%) showed an increase in MoCA score, 35 (31.5%) showed a decrease, and 17 (15.3%) remained unchanged (Figure 2B).

**TABLE 2:** MoCA‐based cognitive outcomes after STN‐DBS.

**Panel A. Overall and baseline cognitive-status groups**						

**Comparison**	***n***	**Preoperative**	**Postoperative**	**Change**	**p**	**Effect size *r***

Overall MoCA	111	19.2 ± 4.8	20.1 ± 5.7	+0.9 ± 4.5	0.012	0.238
MoCA‐defined MCI, MoCA	73	16.4 ± 3.3	18.1 ± 5.3	+1.7 ± 4.6	0.003	0.350
Normal baseline cognition, MoCA	38	24.4 ± 2.4	23.9 ± 4.2	−0.5 ± 3.8	0.906	0.019

**Panel B. Exploratory subgroup analyses of MoCA change**						

**Subgroup**	***n***	**Preoperative MoCA**	**Postoperative MoCA**	**Change**	**p**	**Effect size *r***

Age ≤ 60 years	37	20.2 ± 4.8	20.4 ± 6.6	+0.2 ± 4.7	0.274	0.180
Age 61–70 years	54	18.0 ± 4.9	19.5 ± 5.1	+1.4 ± 4.1	0.017	0.325
Age > 70 years	15	20.1 ± 4.3	22.0 ± 5.2	+1.9 ± 5.3	0.232	0.309
Primary education, ≤ 6 years	33	16.5 ± 4.2	16.4 ± 6.4	−0.1 ± 5.7	0.860	0.031
Middle education, 7–9 years	44	19.7 ± 4.6	20.9 ± 4.4	+1.2 ± 3.5	0.028	0.332
High education, ≥ 10 years	29	21.7 ± 4.0	23.0 ± 4.4	+1.3 ± 4.2	0.091	0.313
Male	62	20.1 ± 4.4	21.4 ± 5.2	+1.3 ± 4.8	0.013	0.314
Female	48	18.1 ± 5.2	18.4 ± 5.9	+0.4 ± 4.0	0.534	0.090
Disease duration ≤ 7 years	47	20.7 ± 4.8	21.7 ± 5.4	+1.1 ± 4.5	0.018	0.345
Disease duration 8–12 years	38	17.8 ± 4.7	18.0 ± 5.6	+0.2 ± 4.6	0.774	0.047
Disease duration > 12 years	20	17.4 ± 3.7	19.6 ± 5.8	+2.1 ± 4.2	0.029	0.490

**Panel C. Rates of MoCA decline**						

**Decline threshold**	**Overall**	**MoCA-defined MCI**	**Normal cognition**		**p**	

MoCA decline ≥ 1 point	35/111 (31.5%)	20/73 (27.4%)	15/38 (39.5%)		0.205	
MoCA decline ≥ 2 points	26/111 (23.4%)	16/73 (21.9%)	10/38 (26.3%)		0.641	
MoCA decline ≥ 3 points	19/111 (17.1%)	12/73 (16.4%)	7/38 (18.4%)		0.796	

*Note:* Values are mean ± SD or *n*/*N* (%), unless otherwise indicated. Pre‐ and postoperative scores were compared using the Wilcoxon signed‐rank test. Effect size was calculated as $r=\left|Z\right|/\sqrt{N}$. MoCA‐defined MCI was operationally defined as baseline MoCA ≤ 21. Subgroup analyses were exploratory and were not adjusted for multiple comparisons. MoCA, Montreal Cognitive Assessment; STN‐DBS, subthalamic nucleus deep brain stimulation.

Abbreviation: MCI, mild cognitive impairment.

**FIGURE 2:**
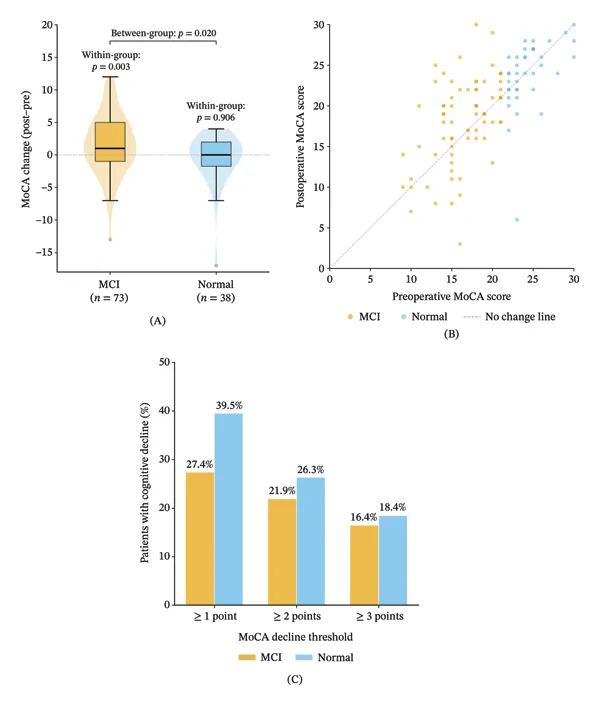
MoCA‐based cognitive outcomes after STN‐DBS. (A) Distribution of postoperative MoCA change according to baseline cognitive status. (B) Paired preoperative and postoperative MoCA scores in the overall cohort; the dashed diagonal line indicates no change. (C) Rates of MoCA decline according to different decline thresholds. For brevity, group labels in the figure are shown as MCI and Normal. MCI refers to MoCA‐defined mild cognitive impairment, operationally defined as preoperative MoCA ≤ 21; Normal refers to normal baseline cognition, defined as preoperative MoCA > 21. Abbreviations: MCI, mild cognitive impairment; MoCA, Montreal Cognitive Assessment; STN‐DBS, subthalamic nucleus deep brain stimulation.

### 3.4. Cognitive Outcomes by Baseline Cognitive Status

In unadjusted analyses stratified by baseline cognitive status, the MoCA‐defined MCI group showed a modest postoperative increase in MoCA score (+1.7 ± 4.6; *p* = 0.003; *r* = 0.350), whereas the normal‐cognition group showed no significant change (−0.5 ± 3.8; *p* = 0.906; *r* = 0.019). The unadjusted change differed between groups (*p* = 0.020, Mann–Whitney *U* test) (Figure 2A). Because cognitive‐status groups were defined from baseline MoCA and change scores may be influenced by baseline‐score dependence and regression to the mean, these comparisons were interpreted descriptively. Baseline‐adjusted analyses of postoperative MoCA are reported in Section 3.7.

Sensitivity analyses using alternative baseline MoCA cutoffs showed a similar descriptive pattern. When lower baseline cognition was defined as MoCA ≤ 20, patients below the cutoff showed a modest postoperative MoCA increase (+1.8 ± 4.9; *p* = 0.004), whereas those above the cutoff remained stable (−0.3 ± 3.5; *p* = 0.872; between‐group *p* = 0.017). A similar pattern was observed using the ≤ 22 cutoff and the education‐adjusted MoCA classification (Supporting Table S3). These sensitivity analyses indicate that the unadjusted pattern was not specific to the prespecified cutoff of 21, but they do not exclude baseline‐score effects or regression to the mean.

### 3.5. Subgroup Analyses

Exploratory subgroup analyses of MoCA change are summarized in Table 2. Nominal postoperative MoCA increases were observed in patients aged 61–70 years (Δ = +1.4 ± 4.1; *p* = 0.017), men (Δ = +1.3 ± 4.8; *p* = 0.013), patients with disease duration ≤ 7 years (Δ = +1.1 ± 4.5; *p* = 0.018), and patients with disease duration > 12 years (Δ = +2.1 ± 4.2; *p* = 0.029). Among education strata, the middle school group also showed a nominal increase (Δ = +1.2 ± 3.5; *p* = 0.028). These findings should be interpreted descriptively because the analyses were exploratory, unadjusted for multiple comparisons, and not supported by formal interaction testing.

### 3.6. Rates of Cognitive Decline

Clinically meaningful cognitive decline, defined as a MoCA decrease of at least 2 points, occurred in 26 of 111 patients (23.4%). Rates were similar in the MoCA‐defined MCI and normal cognition groups (21.9% vs. 26.3%; *p* = 0.641). With a more stringent decline threshold of at least 3 points, 19 patients (17.1%) met the criterion, including 16.4% of patients with MoCA‐defined MCI and 18.4% of those with normal cognition. Decline rates across thresholds are shown in Table 2 and Figure 2C.

### 3.7. Predictors of Postoperative Cognitive Performance and Cognitive Decline

Multivariable baseline‐adjusted linear regression was performed in 90 patients with complete covariate data. The overall model was statistically significant (*R* ^2^ = 0.528, adjusted *R* ^2^ = 0.482; *p* < 0.001). Higher baseline MoCA scores (*B* = 0.469, SE = 0.131, 95% CI 0.209–0.729; *p* < 0.001) and more years of education (*B* = 0.514, SE = 0.144, 95% CI 0.227–0.801; *p* < 0.001) were independently associated with higher postoperative MoCA scores. Age, sex, disease duration, UPDRS‐III medication‐off score, LEDD, and Beck Depression Inventory score were not significantly associated with postoperative MoCA performance (Table 3; Figure 3A).

**TABLE 3:** Predictors of postoperative cognitive performance and clinically meaningful cognitive decline after STN‐DBS.

**Panel A. Multiple linear regression for postoperative MoCA score**				

**Variable**	***B***	**SE**	**95% CI**	**p**

Baseline MoCA	0.469	0.131	(0.209, 0.729)	< 0.001
Age at surgery, years	0.028	0.060	(−0.091, 0.148)	0.637
Sex, male = 1	0.344	1.012	(−1.670, 2.358)	0.735
Disease duration, years	−0.066	0.103	(−0.271, 0.138)	0.520
UPDRS‐III, medication‐off	−0.021	0.027	(−0.075, 0.033)	0.447
LEDD, mg/day	−0.001	0.001	(−0.004, 0.001)	0.283
BECK depression score	−0.026	0.053	(−0.130, 0.079)	0.628
Education, years	0.514	0.144	(0.227, 0.801)	< 0.001

**Panel B. Full logistic regression for clinically meaningful cognitive decline**				

**Variable**	**OR**	**95% CI**	**p**	

Baseline MoCA	1.224	(1.025, 1.463)	0.026	
Age at surgery, years	1.027	(0.955, 1.104)	0.477	
Sex, male = 1	1.254	(0.354, 4.441)	0.725	
Disease duration, years	0.989	(0.861, 1.136)	0.879	
UPDRS‐III, medication‐off	1.009	(0.976, 1.042)	0.603	
LEDD, mg/day	1.001	(1.000, 1.003)	0.182	
BECK depression score	1.022	(0.960, 1.087)	0.502	
Education, years	0.751	(0.615, 0.916)	0.005	

**Panel C. Parsimonious logistic model**				

**Variable**	**OR**	**95% CI**	**p**	

Baseline MoCA	1.160	(1.007, 1.336)	0.039	
Education, years	0.776	(0.657, 0.916)	0.003	
Age at surgery, years	1.033	(0.966, 1.105)	0.339	

*Note:* Dependent variable: postoperative MoCA score; *n* = 90; *R* ^2^ = 0.528; adjusted *R* ^2^ = 0.482; model *p* < 0.001. Dependent variable: MoCA decline ≥ 2 points; *n* = 90; events = 20; pseudo‐*R* ^2^ = 0.128; LR *p* = 0.142. Dependent variable: MoCA decline ≥ 2 points; predictors prespecified as baseline MoCA, education, and age; *n* = 101; events = 22; pseudo‐*R* ^2^ = 0.101; LR *p* = 0.013. *B* values are unstandardized coefficients. ORs are reported per one‐unit increase unless otherwise specified. The full logistic model should be interpreted cautiously because only 20 cognitive‐decline events were available. The parsimonious model was used to reduce overfitting risk. BECK, Beck Depression Inventory; MoCA, Montreal Cognitive Assessment; STN‐DBS, subthalamic nucleus deep brain stimulation; UPDRS‐III, Unified Parkinson’s Disease Rating Scale Part III.

Abbreviations: CI, confidence interval; LEDD, levodopa equivalent daily dose; OR, odds ratio.

**FIGURE 3:**
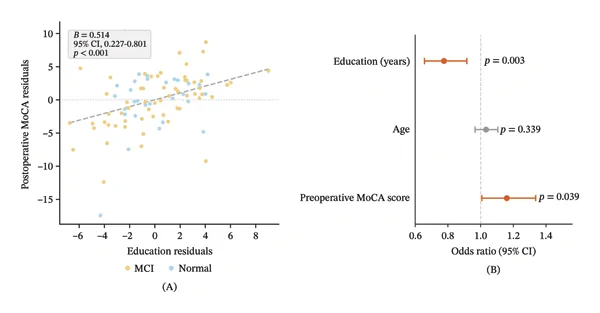
Adjusted associations with postoperative cognitive performance and clinically meaningful MoCA decline. (A) Partial regression plot showing the adjusted association between years of education and postoperative MoCA score after adjustment for baseline MoCA, age at surgery, sex, disease duration, medication‐off UPDRS‐III score, levodopa equivalent daily dose, and Beck Depression Inventory score. Points are colored according to baseline MoCA‐defined cognitive status for visualization; the dashed line represents the overall adjusted association. (B) Forest plot showing predictors of clinically meaningful cognitive decline, defined as a MoCA decrease of at least 2 points, in the parsimonious logistic regression model including baseline MoCA, years of education, and age at surgery. Points represent odds ratios, and horizontal lines represent 95% confidence intervals. The vertical dashed line indicates an odds ratio of 1. Orange indicates statistically significant associations (*p* < 0.05), whereas gray indicates a nonsignificant association. Abbreviations: BECK, Beck Depression Inventory; CI, confidence interval; LEDD, levodopa equivalent daily dose; MCI, mild cognitive impairment; MoCA, Montreal Cognitive Assessment; OR, odds ratio; UPDRS‐III, the Unified Parkinson’s Disease Rating Scale Part III.

In a separate baseline‐adjusted sensitivity analysis, the MoCA‐defined cognitive‐status group did not provide additional explanatory information beyond the baseline MoCA score (*B* = 0.683, 95% CI −2.099–3.465; *p* = 0.627). This finding supports a descriptive interpretation of the greater unadjusted MoCA increase observed in the lower‐baseline group rather than evidence of an independent cognitive‐status group effect.

In the full logistic regression model, higher educational attainment was associated with lower odds of clinically meaningful MoCA decline (OR = 0.751, 95% CI 0.615–0.916; *p* = 0.005). Higher baseline MoCA was also associated with greater odds of meeting the ≥ 2‐point decline threshold (OR = 1.224, 95% CI 1.025–1.463; *p* = 0.026) (Table 3, Panel B). In the parsimonious model, education remained associated with lower odds of decline (OR = 0.776, 95% CI 0.657–0.916; *p* = 0.003), while higher baseline MoCA remained associated with greater odds of meeting the decline threshold (OR = 1.160, 95% CI 1.007–1.336; *p* = 0.039). Age was not significantly associated with decline (Table 3, Panel C; Figure 3B). Because the decline outcome was defined relative to baseline MoCA, associations involving baseline MoCA were interpreted cautiously.

Additional sensitivity analyses accounting for postoperative cognitive assessment timing yielded materially similar results. Education remained associated with higher postoperative MoCA performance after adjustment for follow‐up duration (*B* = 0.514, *p* < 0.001) and with lower odds of clinically meaningful MoCA decline in the parsimonious logistic model (OR = 0.772, *p* = 0.003). Follow‐up duration was associated with neither postoperative MoCA performance (*p* = 0.963) nor clinically meaningful decline (*p* = 0.686). The MoCA‐defined cognitive‐status group effect also remained nonsignificant after additional adjustment for follow‐up duration (*p* = 0.587) (Supporting Table S4).

## 4. Discussion

In this retrospective cohort of patients with PD undergoing bilateral STN‐DBS, group‐level MoCA‐based global cognitive screening performance did not deteriorate. Although patients with lower baseline MoCA scores showed a modest increase in unadjusted analyses, this pattern was interpreted descriptively because change scores may be influenced by baseline‐score dependence and regression to the mean. In the baseline‐adjusted linear model, higher baseline MoCA and more years of education were independently associated with higher postoperative MoCA scores, while higher educational attainment was also associated with lower odds of clinically meaningful MoCA decline in both logistic models. Importantly, additional adjustment for postoperative cognitive assessment timing did not materially alter these associations, and follow‐up duration itself was not associated with postoperative MoCA performance or clinically meaningful decline. These findings suggest that education may provide useful adjunctive information regarding postoperative screening‐level cognitive performance and decline risk, but they should not be interpreted as evidence of cognitive enhancement or broad neuropsychological recovery.

Patients with MoCA‐defined MCI were not at higher risk of meeting the prespecified ≥ 2‐point MoCA decline threshold than patients with normal baseline cognition, which is broadly consistent with a previous MoCA‐based cohort study reporting no excess postoperative cognitive risk in patients with PD‐MCI [9]. Recent studies have likewise suggested that carefully selected patients with preoperative cognitive impairment may still derive motor and functional benefit from DBS [17–19]. However, the present findings should not be interpreted as evidence that baseline cognitive impairment is protective or that STN‐DBS improves cognition. The greater unadjusted MoCA increase observed in the lower‐baseline group showed a similar descriptive pattern across alternative MoCA cutoffs and the education‐adjusted classification, indicating that the finding was not specific to the prespecified cutoff of 21. Nevertheless, because these subgroup definitions were derived from baseline MoCA, the sensitivity analyses do not eliminate baseline‐score dependence, regression to the mean, practice effects, possible influence of postoperative motor improvement on test performance, potential floor effects in individual MoCA tasks, or measurement variability. Consistent with this interpretation, the MoCA‐defined cognitive‐status group did not provide additional explanatory information beyond continuous baseline MoCA in the baseline‐adjusted analysis, and this result was unchanged after additional adjustment for follow‐up duration. Accordingly, the subgroup findings are best interpreted as showing no excess risk of global screening‐level decline in the MoCA‐defined MCI group, rather than evidence of postoperative cognitive benefit.

Beyond baseline‐score effects, interpretation is also constrained by the use of total MoCA as the sole cognitive outcome. Cognitive changes after STN‐DBS may be domain‐specific rather than global, with verbal fluency, processing speed, executive function, and delayed recall among the domains most consistently reported as vulnerable [4–7]. Domain‐level MoCA subscores were not systematically available in the retrospective records; complete paired pre‐ and postoperative domain‐level data were available for only three patients in the final cohort, precluding a reliable analysis of specific cognitive domains. Therefore, stability or modest improvement in total MoCA should not be interpreted as preservation or improvement across all cognitive domains. More recent neuropsychological and prediction studies likewise suggest that postoperative cognitive risk may be missed by global screening instruments alone [20–22]. Without comprehensive domain‐level neuropsychological testing or a nonsurgical control group, the present study cannot distinguish true cognitive change from test–retest effects, practice effects, or natural within‐person variability.

Education emerged as the most consistent reserve‐related marker in our analyses. In the baseline‐adjusted linear model, each additional year of education was associated with a higher postoperative MoCA score, independent of baseline MoCA and other clinical covariates. Higher educational attainment was also associated with lower odds of clinically meaningful MoCA decline in both the full and parsimonious logistic models. These associations remained materially unchanged after additional adjustment for postoperative follow‐up duration, reducing concern that heterogeneous assessment timing accounted for the observed education‐related findings. This pattern is compatible with the cognitive reserve hypothesis, which proposes that individuals with greater reserve, partly reflected by educational attainment, may better maintain cognitive performance despite underlying neuropathological burden. Longitudinal studies in PD have similarly linked higher cognitive reserve with better cognitive performance and slower cognitive decline. Our findings therefore suggest that education may provide adjunctive information regarding postoperative cognitive resilience after STN‐DBS, although the observational design does not establish a causal protective effect.

Not all DBS cohorts have identified education as a predictor of postoperative cognitive outcome. One previous MoCA‐based cohort did not observe such an association [9], whereas another Chinese PD‐DBS cohort reported that education independently predicted postoperative MoCA improvement [16]. These differences may reflect variation in cohort composition, educational distribution, cognitive assessment methods, and follow‐up timing. Education should therefore be regarded as a readily available proxy for cognitive reserve rather than a standalone measure of cognitive resilience or an independent basis for patient selection. In clinical practice, educational history may provide useful adjunctive information. It should be interpreted alongside baseline cognitive performance, age, mood, disease characteristics, motor phenotype, surgical goals, family support, patient preference, and comprehensive neuropsychological assessment. Patients with fewer years of formal education may warrant more detailed counseling, closer postoperative cognitive surveillance, and a lower threshold for additional cognitive assessment, but education alone should not determine DBS candidacy.

At the same time, preservation of group‐level screening performance should not obscure individual risk. Twenty‐six patients (23.4%) met the prespecified ≥ 2‐point threshold for clinically meaningful MoCA decline. This rate falls within the range of previous reports, although it was lower than the rates reported in two prior STN‐DBS cohorts [23, 24]. Cross‐study differences in follow‐up duration, decline thresholds, and assessment instruments may partly account for these discrepancies. Decline rates did not differ significantly between the MoCA‐defined MCI and normal‐cognition groups, indicating that postoperative decline was not confined to patients with lower baseline screening scores. Clinically, a normal preoperative MoCA score therefore does not eliminate the possibility of postoperative decline, supporting cognitive surveillance across baseline cognitive‐status groups.

Earlier studies have identified several potential predictors of cognitive decline after STN‐DBS. Older age and poorer preoperative neuropsychological performance are among the more consistently reported clinical risk markers [25, 26], while surgical and anatomical factors, including electrode trajectory, stimulation location, and structural vulnerability of cognitive networks, may also contribute to postoperative cognitive outcomes [27–29]. More recent work further suggests that baseline domain‐level cognitive profiles may improve prediction of subsequent decline [30]. These factors could not be fully evaluated in the present study because cognitive outcomes were limited to MoCA‐based screening and detailed anatomical and domain‐level neuropsychological data were unavailable. In our multivariable analyses, beyond baseline MoCA and education, the other measured clinical covariates were not consistently associated with postoperative cognitive outcomes. These null findings should be interpreted cautiously given the modest complete‐case sample and the limited number of cognitive‐decline events, which reduced power to detect weaker associations.

Several limitations should be acknowledged. The retrospective, single‐center design precludes causal inference and leaves room for selection bias. A substantial proportion of the original surgical cohort lacked paired postoperative MoCA data, although baseline cognitive scores and education were similar between included and excluded patients. Postoperative cognitive assessments were obtained during routine clinical follow‐up rather than at a fixed protocol‐defined time point. Although the median follow‐up interval was approximately 11 months, follow‐up duration varied across individuals. Importantly, follow‐up duration was similar between baseline cognitive‐status groups, and additional adjustment for follow‐up duration did not materially alter the main findings. Nevertheless, residual effects of assessment timing cannot be fully excluded. Earlier assessments may be influenced by postoperative recovery, medication adjustment, DBS programming, and practice effects, whereas later assessments may increasingly reflect disease progression. MCI was defined using a MoCA cutoff rather than formal MDS PD‐MCI criteria or comprehensive domain‐level testing. Thus, the subgroup results apply to MoCA‐defined MCI, not clinically adjudicated PD‐MCI. Similarly, the MoCA is a global cognitive screening instrument and may miss domain‐specific changes, particularly in verbal fluency and executive function, which are central concerns in DBS neuropsychology [6, 7]. The number of decline events was also limited. Genetic information, including GBA1 status, was unavailable, although recent studies suggest that genetic background may influence cognitive outcomes after DBS [31, 32]. Finally, education is only one proxy for cognitive reserve and does not capture occupational complexity, literacy quality, lifelong cognitive activity, or social engagement.

The clinical implications should therefore be interpreted cautiously. MoCA‐defined MCI alone should not automatically rule out STN‐DBS when multidisciplinary evaluation supports candidacy, but this finding should not be interpreted as a reason to relax neuropsychological screening. Rather, low MoCA, education, mood, age, expected motor benefit, family support, and patient goals should be considered together. Prospective multicenter studies with standardized neuropsychological batteries, fixed follow‐up windows, and richer measures of cognitive reserve are needed to determine the clinical significance of these screening‐level findings.

## 5. Conclusion

In this retrospective cohort of 111 patients with PD undergoing bilateral STN‐DBS, group‐level MoCA‐based global cognitive screening performance showed no overall deterioration. Although patients with lower baseline MoCA scores showed a modest unadjusted postoperative increase, the baseline cognitive‐status group did not provide additional explanatory information after adjustment for baseline MoCA, supporting a cautious descriptive interpretation of this finding. Higher educational attainment was independently associated with better postoperative MoCA performance and lower odds of clinically meaningful MoCA decline, and the education‐related associations remained materially unchanged in sensitivity analyses that additionally adjusted for postoperative follow‐up duration. Education may therefore provide useful adjunctive information for preoperative counseling and postoperative cognitive monitoring, but it cannot replace comprehensive neuropsychological assessment. Prospective studies using standardized follow‐up intervals and multidomain neuropsychological testing are needed to determine the clinical significance of these screening‐level findings.

## Author Contributions

Siyan Chen: conceptualization, methodology, formal analysis, writing–original draft, writing–review and editing. Yuting Zhang: conceptualization, methodology, formal analysis, writing–original draft, writing–review and editing. Chenchen Cui: data curation, formal analysis. Min Xu: investigation, data curation. Wei Zhang: resources, supervision, project administration, writing–review and editing. Dayong Shen: supervision, project administration, writing–review and editing. Jie Zu: supervision, project administration, writing–review and editing.

## Funding

This research did not receive any specific grant from funding agencies in the public, commercial, or not‐for‐profit sectors.

## Disclosure

All authors approved the final version of the manuscript.

## Ethics Statement

The study was approved by the Ethics Committee of the Affiliated Hospital of Xuzhou Medical University (approval no. XYFY2024‐KL501‐01). The study was conducted in accordance with the Declaration of Helsinki. The requirement for written informed consent was waived by the Ethics Committee because of the retrospective nature of the study and the use of anonymized clinical data. Patient privacy and confidentiality were protected throughout the study.

## Consent

This manuscript does not contain identifiable individual patient data.

## Conflicts of Interest

The authors declare no conflicts of interest.

## General Statement

Permission to use the Chinese (Beijing) paper version of the Montreal Cognitive Assessment (MoCA) for this academic retrospective study was obtained from MoCA Test Inc. Under a signed Academic Permission Agreement. The manuscript reports MoCA‐derived scores only and does not reproduce the MoCA test form or administration/scoring instructions.

## Supporting Information

Additional supporting information can be found online in the Supporting Information section.

## Supporting information


**Supporting Information 1** The supporting information includes four supplementary tables. Supporting Table S1 presents motor outcomes in the broader STN‐DBS surgical cohort. Supporting Table S2 compares baseline characteristics between patients included in the final cognitive analysis and those excluded because of unavailable postoperative MoCA assessment or incomplete cognitive records. Supporting Table S3 reports sensitivity analyses using alternative baseline MoCA cutoffs and an education‐adjusted MoCA classification. Supporting Table S4 presents sensitivity analyses accounting for postoperative cognitive assessment timing.

**Supporting Information 2** STROBE Checklist.

## Data Availability

The data that support the findings of this study are available from the corresponding author upon reasonable request, subject to institutional and ethical restrictions related to patient privacy.
