# Supplementary material for: Education, MoCA‐Defined Baseline Cognitive Status, and Screening‐Level Cognitive Change After Bilateral Subthalamic Nucleus Deep Brain Stimulation in Parkinson’s Disease: A Retrospective Cohort Study
Source: Parkinsons Dis. 2026 Nov 14;2026:1801194. doi: 10.1155/padi/1801194 (PMC13769045; doi:10.1155/padi/1801194)
Supplement: Supplementary file 1 [file PADI-2026-1801194-s001.docx]

**Supplementary material**

**Supplementary Table S1.** Motor outcomes in the broader STN-DBS surgical cohort

| Comparison | n | Preoperative | Postoperative | Improvement (%) | p | Effect size r |
| --- | --- | --- | --- | --- | --- | --- |
| MED-OFF → DBS-ON + MED-ON | 201 | 58.4 ± 16.9 | 25.9 ± 13.1 | 54.8% | <0.001 | 0.862 |
| MED-OFF → DBS-ON + MED-OFF | 51 | 60.0 ± 17.9 | 32.6 ± 13.8 | 43.1% | <0.001 | 0.829 |
| MED-OFF → DBS-OFF + MED-OFF | 201 | 57.9 ± 16.9 | 48.8 ± 19.6 | 14.0% | <0.001 | 0.461 |
| MED-ON → DBS-ON + MED-ON | 197 | 30.0 ± 12.9 | 25.6 ± 12.9 | 7.0% | <0.001 | 0.344 |

UPDRS-III scores were analyzed in patients with available paired motor assessments. Paired sample sizes varied across stimulation and medication conditions. Motor outcomes were reported as descriptive background motor measures and were not included as primary cognitive outcomes. Comparisons were performed using the Wilcoxon signed-rank test. Postoperative DBS-off assessments were performed according to the center’s routine clinical protocol, which included a predefined waiting period after stimulation discontinuation; however, the exact switch-off time and achieved washout duration were not consistently documented in the retrospective records and could not be verified for every assessment. DBS-off comparisons should therefore be interpreted descriptively.

Abbreviations: DBS, deep brain stimulation; MED, medication; STN-DBS, subthalamic nucleus deep brain stimulation; UPDRS-III, Unified Parkinson’s Disease Rating Scale Part III.

**Supplementary Table S2.** Baseline comparison between included and excluded patients

| **Variable** | **Included**  **(n = 111)** | **Excluded**  **(n = 169)** | **Available n** | **p** |
| --- | --- | --- | --- | --- |
| Male sex, n (%) | 62/110 (56.4%) | 89/162 (54.9%) | 110/162 | 0.914 |
| Age at surgery, years | 62.9 ± 8.1 | 64.8 ± 8.4 | 106/130 | 0.073 |
| Disease duration, years | 9.0 ± 4.6 | 9.4 ± 4.3 | 105/132 | 0.223 |
| Surgery year | 2019.0 ± 1.7 | 2021.3 ± 2.3 | 110/165 | <0.001 |
| Education, years | 8.4 ± 4.0 | 7.7 ± 4.7 | 106/156 | 0.173 |
| LEDD, mg/day | 772.8 ± 342.0 | 757.9 ± 334.6 | 101/150 | 0.936 |
| Hoehn–Yahr stage, medication-off | 2.9 ± 0.7 | 2.9 ± 0.7 | 66/72 | 0.806 |
| Baseline MoCA | 19.2 ± 4.8 | 19.1 ± 5.6 | 111/142 | 0.812 |
| UPDRS-III, medication-off | 58.0 ± 18.7 | 61.0 ± 16.4 | 106/152 | 0.206 |
| UPDRS-III, medication-on | 28.5 ± 13.1 | 33.0 ± 13.1 | 104/148 | 0.004 |
| Levodopa response, % | 51.3 ± 14.1 | 45.9 ± 17.5 | 104/147 | 0.008 |
| BECK depression score | 19.3 ± 10.8 | 20.0 ± 10.5 | 108/143 | 0.512 |
| PDQ-39 score | 61.8 ± 27.1 | 65.9 ± 29.7 | 106/151 | 0.286 |
| Education level, n (%) |  |  | 106/156 | 0.222 |
| Primary, ≤6 years | 33 (31.1%) | 65 (41.7%) |  |  |
| Middle school, 7–9 years | 44 (41.5%) | 54 (34.6%) |  |  |
| High school or above, ≥10 years | 29 (27.4%) | 37 (23.7%) |  |  |

Values are presented as mean ± SD or n (%), unless otherwise indicated. Included patients were defined as those with both preoperative and postoperative MoCA data; excluded patients were those without paired postoperative MoCA data or with incomplete cognitive records. Available n indicates the number of patients with non-missing data in the included and excluded groups, respectively. p values were calculated using the Mann–Whitney U test for continuous variables and the chi-square test for categorical variables.

Abbreviations: BECK, Beck Depression Inventory; LEDD, levodopa equivalent daily dose; MoCA, Montreal Cognitive Assessment; PDQ-39, Parkinson’s Disease Questionnaire-39; UPDRS-III, Unified Parkinson’s Disease Rating Scale Part III.

**Supplementary Table S3.** Sensitivity analyses using alternative MoCA cutoffs for baseline cognitive status

| **Definition** | **Group** | **n** | **Pre-op**  **MoCA** | **Post-op**  **MoCA** | **MoCA change** | **Within-group p** | **Effect size r** | **Decline ≥2** | **Between-group p** |
| --- | --- | --- | --- | --- | --- | --- | --- | --- | --- |
| Raw baseline MoCA cutoff 20 | ≤20 | 63 | 15.7 ± 2.9 | 17.5 ± 5.4 | +1.8 ± 4.9 | 0.004 | 0.361 | 14 (22.2%) | 0.017 |
|  | >20 | 48 | 23.7 ± 2.6 | 23.4 ± 4.0 | -0.3 ± 3.5 | 0.872 | 0.023 | 12 (25.0%) |  |
| Raw baseline MoCA cutoff 21 | ≤21 | 73 | 16.4 ± 3.3 | 18.1 ± 5.3 | +1.7 ± 4.6 | 0.003 | 0.350 | 16 (21.9%) | 0.020 |
|  | >21 | 38 | 24.4 ± 2.4 | 23.9 ± 4.2 | -0.5 ± 3.8 | 0.906 | 0.019 | 10 (26.3%) |  |
| Raw baseline MoCA cutoff 22 | ≤22 | 82 | 17.0 ± 3.5 | 18.5 ± 5.2 | +1.5 ± 4.5 | 0.003 | 0.326 | 18 (22.0%) | 0.038 |
|  | >22 | 29 | 25.1 ± 2.3 | 24.4 ± 4.5 | -0.7 ± 4.1 | 0.774 | 0.053 | 8 (27.6%) |  |
| Education-adjusted baseline MoCA cutoff 21 | ≤21 | 60 | 15.9 ± 3.0 | 17.6 ± 5.6 | +1.7 ± 4.9 | 0.008 | 0.343 | 14 (23.3%) | 0.024 |
|  | >21 | 46 | 23.6 ± 2.4 | 23.3 ± 4.0 | -0.3 ± 3.5 | 0.918 | 0.015 | 11 (23.9%) |  |

Values are mean ± SD or n (%), unless otherwise indicated. Within-group comparisons used the Wilcoxon signed-rank test; between-group comparisons used the Mann-Whitney U test. Between-group p values refer to comparisons of MoCA change between the two groups within each sensitivity definition. For the education-adjusted classification, one point was added to the baseline MoCA score for patients with ≤12 years of education, capped at 30 points; patients with missing education data were excluded from this sensitivity analysis (n = 106). MoCA change was calculated from raw MoCA scores in all analyses.

Abbreviations: MoCA, Montreal Cognitive Assessment

**Supplementary Table S4.** Sensitivity analyses accounting for postoperative cognitive assessment timing

**Panel A. Baseline-adjusted cognitive-status sensitivity model including follow-up duration**

*Dependent variable: postoperative MoCA score; n = 111; R² = 0.425; adjusted R² = 0.409; model p < 0.001.*

| **Variable** | **B** | **SE** | **95% CI** | **p** |
| --- | --- | --- | --- | --- |
| Baseline MoCA | 0.826 | 0.140 | (0.549, 1.104) | <0.001 |
| MoCA-defined MCI group | 0.770 | 1.415 | (-2.034, 3.575) | 0.587 |
| Follow-up duration, months | -0.047 | 0.076 | (-0.196, 0.103) | 0.539 |

**Panel B. Baseline-adjusted multivariable linear model including follow-up duration**

*Dependent variable: postoperative MoCA score; n = 90; R² = 0.528; adjusted R² = 0.475; model p < 0.001.*

| **Variable** | **B** | **SE** | **95% CI** | **p** |
| --- | --- | --- | --- | --- |
| Baseline MoCA | 0.470 | 0.132 | (0.207, 0.732) | <0.001 |
| Age at surgery, years | 0.028 | 0.060 | (-0.092, 0.148) | 0.638 |
| Sex, male = 1 | 0.339 | 1.024 | (-1.699, 2.377) | 0.741 |
| Disease duration, years | -0.066 | 0.104 | (-0.274, 0.142) | 0.530 |
| UPDRS-III, medication-off | -0.021 | 0.027 | (-0.076, 0.034) | 0.449 |
| LEDD, mg/day | -0.001 | 0.001 | (-0.004, 0.001) | 0.287 |
| BECK depression score | -0.026 | 0.053 | (-0.131, 0.080) | 0.631 |
| Education, years | 0.514 | 0.145 | (0.225, 0.803) | <0.001 |
| Follow-up duration, months | 0.005 | 0.108 | (-0.209, 0.219) | 0.963 |

**Panel C. Parsimonious logistic model including follow-up duration**

*Dependent variable: MoCA decline ≥2 points; n = 101; events = 22; pseudo-R² = 0.103; LR p = 0.028.*

| **Variable** | **OR** | **95% CI** | **p** |
| --- | --- | --- | --- |
| Baseline MoCA | 1.163 | (1.009, 1.342) | 0.038 |
| Education, years | 0.772 | (0.651, 0.914) | 0.003 |
| Age at surgery, years | 1.033 | (0.966, 1.105) | 0.346 |
| Follow-up duration, months | 1.020 | (0.926, 1.124) | 0.686 |

B values are unstandardized regression coefficients. ORs are reported per one-unit increase unless otherwise specified. Follow-up duration was defined as the interval from DBS surgery to the postoperative MoCA assessment used for analysis. In Panel A, the MoCA-defined MCI group was coded as 1, with normal baseline cognition as the reference group. Panel A evaluates whether baseline cognitive-status group remained associated with postoperative MoCA after adjustment for baseline MoCA and follow-up duration. Panel B adds follow-up duration to the primary baseline-adjusted multivariable linear model. Panel C adds follow-up duration to the parsimonious logistic model; the full logistic model was not extended for this sensitivity analysis to limit overfitting given the number of cognitive-decline events. Complete-case analysis was used for each model.

Abbreviations: BECK, Beck Depression Inventory; CI, confidence interval; LEDD, levodopa equivalent daily dose; MCI, mild cognitive impairment; MoCA, Montreal Cognitive Assessment; OR, odds ratio; UPDRS-III, Unified Parkinson's Disease Rating Scale Part III.
