# Supplementary material for: Education, MoCA‐Defined Baseline Cognitive Status, and Screening‐Level Cognitive Change After Bilateral Subthalamic Nucleus Deep Brain Stimulation in Parkinson’s Disease: A Retrospective Cohort Study
Source: Parkinsons Dis. 2026 Nov 14;2026:1801194. doi: 10.1155/padi/1801194 (PMC13769045; doi:10.1155/padi/1801194)
Supplement: Supplementary file 2 [file PADI-2026-1801194-s002.docx]

**STROBE Statement—Checklist of items that should be included in reports of cohort studies**

| **Section/topic** | **Item No.** | **Recommendation** | **Reported in revised manuscript** |
| --- | --- | --- | --- |
| Title and abstract | 1a | Indicate the study’s design with a commonly used term in the title or the abstract | Title: the study design is indicated as “A Retrospective Cohort Study.” Abstract/Methods: retrospective cohort design, STN-DBS cohort, paired pre- and postoperative MoCA data, baseline MoCA-defined cognitive-status grouping, and MoCA-based outcomes. |
| Title and abstract | 1b | Provide in the abstract an informative and balanced summary of what was done and what was found | Abstract: structured Background, Methods, Results, and Conclusions summarize the study rationale, retrospective cohort, MoCA-defined cognitive-status groups, unadjusted MoCA change, baseline-adjusted postoperative MoCA performance, clinically meaningful decline, follow-up-duration sensitivity analyses, and cautious conclusions. |
| Introduction: Background/rationale | 2 | Explain the scientific background and rationale for the investigation being reported | Introduction: first three paragraphs describe PD-related cognitive impairment, uncertainty of STN-DBS cognitive outcomes, MoCA-defined MCI, education, and the cognitive-reserve rationale. |
| Introduction: Objectives | 3 | State specific objectives, including any prespecified hypotheses | Introduction: final paragraph states the objectives and hypothesis regarding baseline MoCA-defined cognitive status, education, postoperative MoCA performance, and clinically meaningful MoCA decline after STN-DBS. |
| Methods: Study design | 4 | Present key elements of study design early in the paper | Methods 2.1: single-center retrospective cohort design, study setting, study period, ethics approval, Declaration of Helsinki compliance, and STROBE reporting are described. |
| Methods: Setting | 5 | Describe the setting, locations, and relevant dates, including periods of recruitment, exposure, follow-up, and data collection | Methods 2.1 and 2.4: Department of Neurology/Parkinson’s Disease Center, The Affiliated Hospital of Xuzhou Medical University, Xuzhou, China; January 2015 to December 2024; preoperative MoCA within 1 month before surgery and postoperative MoCA at least 3 months after surgery. The analytic cohort had a median postoperative MoCA follow-up of 11.4 months (IQR 10.4–13.3; range 5.2–40.8). |
| Methods: Participants | 6a | Give the eligibility criteria, and the sources and methods of selection of participants. Describe methods of follow-up | Methods 2.2 and 2.4: eligibility criteria, exclusion criteria, data source, and follow-up cognitive assessment are described. Results 3.1 and Figure 1 report patient selection and numbers included/excluded. |
| Methods: Participants | 6b | For matched studies, give matching criteria and number of exposed and unexposed | Not applicable. This was not a matched cohort study. |
| Methods: Variables | 7 | Clearly define all outcomes, exposures, predictors, potential confounders, and effect modifiers. Give diagnostic criteria, if applicable | Methods 2.4–2.6: cognitive, motor, mood, patient-reported, education, and follow-up-duration variables; primary and secondary outcomes; MoCA-defined MCI/normal cognition; sensitivity definitions; candidate predictors and covariates are defined. |
| Methods: Data sources/measurement | 8 | For each variable of interest, give sources of data and details of methods of assessment (measurement). Describe comparability of assessment methods if there is more than one group | Methods 2.1 and 2.4: data came from medical records at the study center. Methods 2.4 describes MoCA, UPDRS-III, LEDD, Beck Depression Inventory, PDQ-39, education, and postoperative follow-up measurement. Complete paired MoCA domain-level data were available for only three patients, precluding reliable domain-specific analyses. For postoperative DBS-off motor assessments, a predefined waiting period was used clinically, but the exact washout duration could not be verified retrospectively for every assessment. |
| Methods: Bias | 9 | Describe any efforts to address potential sources of bias | Methods 2.2 and 2.6, Results 3.1 and 3.7, Supplementary Tables S2 and S4, and Discussion/Limitations: incomplete/unreliable cognitive data were excluded; included-versus-excluded comparisons assessed selection bias; baseline-adjusted models addressed baseline-score dependence/regression to the mean; follow-up-duration sensitivity analyses assessed timing-related confounding; complete-case regression samples and residual limitations are reported. |
| Methods: Study size | 10 | Explain how the study size was arrived at | Methods 2.1, 2.2, and Results 3.1: all patients who underwent bilateral STN-DBS during the 2015–2024 study period were screened according to the prespecified eligibility criteria; 111 patients with paired pre- and postoperative MoCA data were included in the final cognitive analysis. |
| Methods: Quantitative variables | 11 | Explain how quantitative variables were handled in the analyses. If applicable, describe which groupings were chosen and why | Methods 2.4–2.6: education categories, MoCA-defined cognitive status, alternative MoCA cutoffs, education-adjusted MoCA classification, subgroup categories, continuous follow-up duration, and regression modeling of quantitative variables are described. |
| Methods: Statistical methods | 12a | Describe all statistical methods, including those used to control for confounding | Methods 2.6: Shapiro-Wilk test, Mann-Whitney U test, chi-square/Fisher’s exact test, Wilcoxon signed-rank test, effect size r, baseline-adjusted multiple linear regression for postoperative MoCA, full and parsimonious logistic regression, complete-case analysis, covariates, ORs/coefficients with 95% CIs, and follow-up-duration sensitivity models are described. |
| Methods: Statistical methods | 12b | Describe any methods used to examine subgroups and interactions | Methods 2.6 and Results 3.5: exploratory subgroup analyses by baseline cognitive status, age group, education level, sex, and disease duration are described; no formal interaction testing was performed. |
| Methods: Statistical methods | 12c | Explain how missing data were addressed | Methods 2.6, Table 1 footnote, Table 3, and Supplementary Table S2: included-versus-excluded comparison, variable denominators, available-n reporting, and complete-case regression sample sizes are provided. |
| Methods: Statistical methods | 12d | If applicable, explain how loss to follow-up was addressed | Results 3.1, Figure 1, Supplementary Table S2, and Discussion/Limitations: exclusions for unavailable postoperative MoCA or incomplete cognitive data are reported; included-versus-excluded comparisons assess potential selection bias. Among included patients, postoperative cognitive follow-up duration is characterized and compared between baseline cognitive-status groups. |
| Methods: Statistical methods | 12e | Describe any sensitivity analyses | Methods 2.5–2.6 and Results 3.4/3.7: sensitivity analyses include alternative baseline MoCA cutoffs (≤20 and ≤22), education-adjusted MoCA classification, a baseline-adjusted cognitive-status model addressing baseline-score dependence/regression to the mean, and models additionally adjusting for follow-up duration. Results are reported in Supplementary Tables S3 and S4. |
| Results: Participants | 13a | Report numbers of individuals at each stage of study—eg numbers potentially eligible, examined for eligibility, confirmed eligible, included in the study, completing follow-up, and analysed | Results 3.1 and Figure 1: 280 patients underwent bilateral STN-DBS, 169 were excluded, and 111 patients with paired pre- and postoperative MoCA data were included in the final cognitive analysis. |
| Results: Participants | 13b | Give reasons for non-participation at each stage | Results 3.1 and Figure 1: exclusions were due to absence of postoperative MoCA assessment (n = 157) or incomplete cognitive data (n = 12). |
| Results: Participants | 13c | Consider use of a flow diagram | Figure 1: Patient selection flowchart; cited in Results 3.1 and described in the Figure 1 legend. |
| Results: Descriptive data | 14a | Give characteristics of study participants (eg demographic, clinical, social) and information on exposures and potential confounders | Results 3.1 and Table 1: demographic, clinical, MoCA, mood, patient-reported, and education variables are summarized overall and by baseline MoCA-defined cognitive status. |
| Results: Descriptive data | 14b | Indicate number of participants with missing data for each variable of interest | Supplementary Table S2 reports available n for individual baseline variables in the included and excluded groups. Table 1 notes that denominators varied because of missing data, and Table 3 reports model-specific complete-case sample sizes. Methods 2.4 additionally reports that complete paired domain-level MoCA data were available for only three patients. |
| Results: Descriptive data | 14c | Summarise follow-up time (eg, average and total amount) | Methods 2.4 and Results 3.1: median time from DBS surgery to postoperative MoCA assessment was 11.4 months (IQR 10.4–13.3; range 5.2–40.8). Follow-up duration was similar between MoCA-defined MCI and normal-cognition groups [11.3 (IQR 10.3–13.2) vs. 11.5 (IQR 10.6–13.5) months; p = 0.666]. The implications of non-fixed timing are discussed in the Limitations. |
| Results: Outcome data | 15 | Report numbers of outcome events or summary measures over time | Results 3.3–3.6, Table 2, and Figure 2: overall and group-level MoCA changes, numbers showing increase/decrease/no change, and MoCA decline event rates are reported. |
| Results: Main results | 16a | Give unadjusted estimates and, if applicable, confounder-adjusted estimates and their precision (eg, 95% confidence interval). Make clear which confounders were adjusted for and why they were included | Results 3.7 and Table 3: baseline-adjusted multiple linear regression estimates postoperative MoCA performance with baseline MoCA and prespecified clinical covariates; full and parsimonious logistic models estimate clinically meaningful decline. Estimates are reported with 95% CIs and p values. Methods 2.6 specifies covariates and model rationale; Supplementary Table S4 shows follow-up-duration-adjusted sensitivity analyses. |
| Results: Main results | 16b | Report category boundaries when continuous variables were categorized | Methods 2.4–2.5 and Table 2: education categories, baseline MoCA-defined MCI cutoff (≤21), normal cognition (>21), alternative MoCA cutoffs, age categories, disease-duration categories, and MoCA decline thresholds are reported. |
| Results: Main results | 16c | If relevant, consider translating estimates of relative risk into absolute risk for a meaningful time period | Results 3.6 and Table 2 Panel C: absolute MoCA decline rates are reported for ≥1-point, ≥2-point, and ≥3-point thresholds. Table 3 reports ORs for clinically meaningful MoCA decline. |
| Results: Other analyses | 17 | Report other analyses done—eg analyses of subgroups and interactions, and sensitivity analyses | Results 3.4–3.7: alternative-cutoff analyses, baseline-adjusted cognitive-status sensitivity analysis, exploratory subgroup analyses, full/parsimonious logistic models, and postoperative-assessment-timing sensitivity analyses are reported. Supplementary Table S3 presents cutoff/education-adjusted analyses and Supplementary Table S4 presents follow-up-duration-adjusted models. |
| Discussion: Key results | 18 | Summarise key results with reference to study objectives | Discussion opening paragraph summarizes key findings in relation to group-level MoCA screening performance, baseline MoCA-defined cognitive status, education, clinically meaningful MoCA decline, and robustness to postoperative assessment timing; Conclusion restates the main findings cautiously. |
| Discussion: Limitations | 19 | Discuss limitations of the study, taking into account sources of potential bias or imprecision. Discuss both direction and magnitude of any potential bias | Discussion/Limitations address the retrospective single-center design, selection bias from missing paired postoperative MoCA data, non-fixed follow-up timing, MoCA-defined rather than formally adjudicated PD-MCI, baseline-score dependence/regression to the mean, practice and potential floor effects, only three patients with complete paired domain-level MoCA data, limited decline events, unavailable genetic data, and education as an incomplete proxy for cognitive reserve. |
| Discussion: Interpretation | 20 | Give a cautious overall interpretation of results considering objectives, limitations, multiplicity of analyses, results from similar studies, and other relevant evidence | Discussion and Conclusion interpret findings cautiously as screening-level associations. Interpretation explicitly considers baseline-score dependence, regression to the mean, practice effects, potential task-level floor effects, domain-specific cognitive risk not captured by total MoCA, non-fixed follow-up timing, lack of a non-surgical control group, exploratory multiplicity, and comparison with prior studies. |
| Discussion: Generalisability | 21 | Discuss the generalisability (external validity) of the study results | Discussion/Limitations and clinical implications address the single-center design and screening-level outcome, and recommend prospective multicenter validation using standardized follow-up intervals, comprehensive multidomain neuropsychological batteries, and broader measures of cognitive reserve. |
| Other information: Funding | 22 | Give the source of funding and the role of the funders for the present study and, if applicable, for the original study on which the present article is based | Declarations: Funding states that the research did not receive any specific grant from public, commercial, or not-for-profit funding agencies. |

*STROBE note: Information should be provided separately for comparison groups where applicable. In this manuscript, the primary comparison groups are MoCA-defined MCI and normal baseline cognition. Reported locations refer to the revised Main Document by section, table, figure, and declaration heading.
